# Bone grafts utilized in dentistry: an analysis of patients' preferences

**DOI:** 10.1186/s12910-015-0044-6

**Published:** 2015-10-20

**Authors:** Ramón Fuentes Fernández, Cristina Bucchi, Pablo Navarro, Víctor Beltrán, Eduardo Borie

**Affiliations:** Research Centre in Dental Sciences, Dental School, Universidad de La Frontera, Manuel Montt #112, 4781176 Temuco, Chile; Mathematics and Statistic Department, Universidad de La Frontera, Temuco, Chile; Department of Dental Materials and Prosthodontics, Dental School of Ribeirão Preto, University of São Paulo, Ribeirão Preto, SP Brazil

**Keywords:** Dentistry, Bone grafts, Dental health surveys, Patient preference

## Abstract

**Background:**

Many procedures currently require the use of bone grafts to replace or recover bone volume that has been resorbed. However, the patient’s opinion and preferences must be taken into account before implementing any treatment. Researchers have focused primarily on assessing the effectiveness of bone grafts rather than on patients' perceptions. Thus, the aim of this study was to explore patients' opinions regarding the different types of bone grafts used in dental treatments.

**Methods:**

One hundred patients were randomly chosen participated in the study. A standardized survey of 10 questions was used to investigate their opinions regarding the different types of bone grafts used in dental treatments. Descriptive statistics were calculated for the different variables, and absolute frequencies and percentages were used as summary measures. A value of p <0.05 was selected as the threshold for statistical significance.

**Results:**

The highest rate of refusal was observed for allografts and xenografts. The grafts with the lowest rates of refusal were autologous grafts (3 %) and alloplastics (2 %). No significant differences were found between the various types of bone grafts in the sociodemographic variables or the refusal/acceptance variable. Similarly, no significant relations were observed between a specific religious affiliation and the acceptance/refusal rates of the various types of graft.

**Conclusions:**

Allografts and xenografts elicited the highest refusal rates among the surveyed patients, and autologous bone and alloplastics were the most accepted bone grafts. Moreover, no differences were found in the sociodemographic variables or religious affiliations in terms of the acceptance/refusal rates of the different bone grafts.

## Background

Currently, many procedures require the use of bone grafts to replace or recover bone volume that has been resorbed due to systemic pathologies [1], periodontal defects [2], tooth loss [3] or other conditions. Advances in modern medicine have led to increases in the availability of new biomaterials that can be used to enhance bone volume recovery. These biomaterials may be obtained from the patient's own body, other humans, animals, or can even be synthetically produced [4]. Despite these advances, little research has been conducted regarding the patients' opinions about the different bone graft materials available or their willingness to use these biomaterials in their surgeries. The source of the bone graft may be objectionable to the patient due to religious, ethical and/or cultural concerns. Some patient perception studies related to medical procedures involving soft-tissue grafts and organ transplants have been conducted [5, 6]; however, in dentistry, such research has primarily focused on assessing the effectiveness of bone grafts rather than on patient perceptions.

The types of bone grafts most frequently used in dentistry include autologous bone grafts, allografts, xenografts and alloplastics. Autologous bone grafts come from donor sites in the patient's own body and have osteoconductive and osteoinductive properties. Such autologous bone grafts also contain osteogenic cells that help reduce the bone healing time [7, 8]. Allografts are another type of bone graft in which the bone is taken from another donor of the same species, and are typically obtained from human cadavers and subsequently subjected to processing [9, 10]. Xenografts consist of bone tissue taken from a different species and have osteoconductive properties and preserve the original bone mineral structure, which is more complex than that of synthetic materials, i.e., alloplastics [11]. Alloplastic bone substitutes may be ceramic, hydroxyapatite, tricalcium phosphate or calcium sulfate [4].

Most clinicians base their decisions primarily on their experience and areas of expertise and often forget to ask the patient’s opinion or explain the advantages and disadvantages of the different procedures and any alternative products that may be used [12]. Such omissions result in a transgression of a main ethical principle, i.e., autonomy. Patients have the right to refuse a specific product or treatment if it is against their philosophical or religious principles. Accordingly, it is mandatory that patients participate in decision-making, that the clinician’s role should be minor and restricted to presenting the current scientific data about the possible and plausible treatment alternatives [12], and that the clinician should listen to the patient’s questions and opinions before a decision regarding the “best alternative” is made. Many procedures and treatments require patients to provide informed consent. This is a patient's ethical and legal right [13] and requires that clinicians describe to the patient the procedure and the origin and type of material to be used in the surgery [14]. The patient's refusal to undergo a treatment proposed by the clinician may prevent the treatment from proceeding and may also become an obstacle to a satisfactory clinician-patient relationship. Therefore, the patient's opinion and preferences must be taken into account before implementing any treatment.

The aim of the present study was to examine patients' opinions regarding the different types of bone grafts used in dental treatments.

## Methods

A survey was administered to 100 dental patients who received treatment at the Dental Clinics of the Universidad of La Frontera in Temuco, Chile between January and July 2014. This sample corresponded to approximately 10 % of the patients treated in our clinics in the course of a year. The patients were chosen randomly. The patients were allocated a number from 1 to 10 in the waiting room, and two numbers were randomly selected each day. The patients were also required to meet the established inclusion criteria of the survey. This research was approved by the Scientific Ethics Committee of Universidad of La Frontera (protocol N. 145/13). The following inclusion criteria were applied:

- adult (18 years of age or older)
- able to read and write
- not under the influence of alcohol or drugs
- had not previously undergone any surgery involving bone graft or bone augmentation

The purpose of the study was briefly explained to the selected patients. Those who agreed to participate in the survey were asked to provide their written free and informed consent.

Before the survey was administered, only the origins of the different types of conventional bone grafts were explained to the patients, and their opinions regarding the acceptance or rejection of each type were acquired.

### Survey

The survey recorded the following participant demographic data:

- gender
- age
- education level: participants were asked to indicate their education level (yes/no). Yes responses were further investigated with the following response options: primary (incomplete/complete), secondary (incomplete/complete) and higher education (incomplete/complete).
- religion: participants were asked, “Do you profess a religious faith?” (yes/no). Yes responses were further investigated by asking the participant to write down the religion they followed.

The survey also included 10 questions as follows:

- Five closed-ended questions (with lists of possible answers) about the level of acceptance of each type of bone graft (acceptance, conditional acceptance or refusal). Respondents were allowed to choose only the single answer that best aligned with their opinion.
- Three open-ended questions (i.e., the patients' spontaneous answers were recorded).
- Two mixed questions that aimed to identify the reasons for refusal (if applicable).

The validity of the survey design and contents were evaluated in November 2013 by a group of experts consisting of three Chilean implantologists and two periodontists at the Universidad of La Frontera's Dental School. Previously, a pilot survey was applied to 10 patients at the University's Dental Clinics, and minor modifications were subsequently made to the questionnaire.

### Statistical analysis

The open and mixed responses were categorized to facilitate the statistical analyses. For example, some patients answered: *“I would not accept this graft because I do not like it”* or *“because it does not seem right to use this graft”* and such responses were classified by the researchers as a “*simple preference”.* Responses such as, *“God does not approve the use of human remains in other humans”* and *“the Bible says…”* were classified as “*religious reasons”.* These questions were for the purposes of the statistical analysis as this research was quantitative and not qualitative in nature. The statistical analyses of the survey results were performed with the SPSS / PC + v.20.0 software (SPSS, Chicago, IL, USA). Descriptive statistics were calculated for the different variables using the absolute frequencies and percentages as the summary measures. The relationships between the categorical variables were estimated with Pearson's chi-square test. A value of p <0.05 was selected as the threshold for statistical significance.

## Results

The total sample was 100 patients. The demographic data of the respondents are presented in Table 1. The majority of the patients surveyed were women (76.8 %) aged 18–30 (44 %) with higher education (60.8 %) who identified as Catholic (54.1 %).

**Table 1:** Demographic data (gender, age group, educational level and religious affiliation) of the surveyed patients (n = 100)

Variable	Categories	Total valid percentage (N = 100 interviewees)
Gender	Men	23.2 %
	Women	76.8 %
Age	18-30	44 %
	31-45	26 %
	46-60	23 %
	61 or older	7 %
Educational level	No formal education	0 %
	Complete or incomplete primary education	1 %
	Complete or incomplete secondary education	37.1 %
	Complete or incomplete higher education	60.8 %
Religious affiliation	No	23.5 %
	Yes	76.5 %
	Catholic	72.6 %
	Evangelical	27.3 %

The type of bone graft that elicited the highest rate of refusal was the allograft; 20 % of the patients stated that under no circumstances would they accept this type of bone graft in their surgery. An additional 21 % said they would only accept an allograft if it was the only option. The reasons given for refusing an allograft were as follows: fear of possible disease transmission (n = 15), the belief that it is wrong to use bone from another human being (n = 17), religious reasons (n = 1) and preferences (n = 3). Statistically significant relationships were observed between gender and the refusal of allografts: women were more likely than men to refuse an allograft and to accept an allograft only as a last resort (0.008).

The type of bone graft that elicited the second-highest refusal rate was the xenograft. 15 % of patients said they would accept this type of bone graft under no circumstances, while 18 % said they would accept a xenograft only as a last resort. The reasons given for refusing a xenograft were as follows: fear of the possible transmission of disease from the animal (n = 16), the belief that it is wrong to use animals for human benefit (n = 12), religious reasons (n = 2) and simple preferences (n = 1).

The types of bone graft that elicited the lowest rates of refusal were alloplastics (2 %) and autologous grafts (3 %) from an intraoral donor site. Interestingly, the patients' reasons for refusing autologous bone grafts were primarily related to the fear of pain, discomfort or possible negative effects on other parts of the body (n = 15). Some patients also refused autologous bone grafts for religious reasons (n = 2) and simple preferences (n = 1).

Table 2 presents additional data about the patients' acceptance or refusal of the different types of bone graft, and the reasons for refusal are presented in Table 3.

**Table 2:** Degrees of patient acceptance of the different types of bone graft (n = 100)

Type of graft	Percentage of validated answers							
	Never	Only as a last resort	Yes, if this type of graft leads to the best results	Yes, if my dentist recommends the use of this type of graft material	Yes. I am comfortable with the use of this graft material.	Only if the animal did not suffer or was killed in order to obtain the graft	Yes, but only from a living donor	Yes, but only from a deceased donor
Alloplastics	2 %	13 %	33 %	23 %	29 %	-	-	-
Autologous bone graft from intraoral donor site (chin or posterior mandible)	3 %	7 %	31 %	20 %	39 %	-	-	-
Autologous bone graft from extraoral donor site (tibia, hip, etc.)	8 %	17 %	26 %	19 %	30 %	-	-	-
Xenograft	15 %	18 %	25 %	15 %	15 %	11 %	-	-
Allograft	20 %	21 %	19 %	0 %	4 %	-	11 %	7 %

**Table 3:** Summary of the reasons for the refusal of the different types of bone graft

Type of graft	Reason for refusal per type of bone graft						
	Fear of possible transmission of disease or infection	Pain, discomfort or fear of affecting another part of the body	It is wrong to use animals for human benefit	It is wrong to use bone material from another human being	Religious reasons	Simple preference	Prefer natural materials
Alloplastics	1 (1 %)	-	-	-	2 (2 %)	2 (2 %)	4 (4 %)
Autologous bone graft from intraoral donor site	-	4 (4 %)	-	-	1 (1 %)	1 (1 %)	-
Autologous bone graft from extraoral donor site	-	11 (11 %)	-	-	1 (1 %)	-	-
Xenograft	16 (16 %)	-	12 (12 %)	-	2 (2 %)	1 (1 %)	-
Allograft	15 (15 %)	-	-	17 (17 %)	1 (1 %)	3 (3 %)	-

No significant differences in the refusal/acceptance rates of the various types of bone graft were observed according to the sociodemographic variables (i.e., age, education level, and adherence to a religious faith). Similarly, no significant relationships between the specific religious affiliation (i.e., Catholic or Evangelical) and the acceptance/refusal rates of the various types of graft were observed.

## Discussion

Bone grafts have made it possible to resolve the problems of the insufficient thickness or height of the jawbone in many patients who require dental implants for either functional or esthetic reasons. Autologous bone is currently considered the “gold standard” for bone regeneration due to its osteoconduction, osteoinduction and osteogenesis-inducing properties [4]. However, autologous bone grafts occasionally have significant drawbacks, such as increased postoperative morbidity, the need for a second surgery and the lack of sufficient bone mass at the donor site [11]. Consequently, science has developed other therapeutic options, such as alloplastic grafts (synthetic bone substitutes), processed bone from species other than that receiving the graft (xenografts), and processed bone from different individuals of the same species (allografts) [4]. Unfortunately, the literature reports some controversial issues regarding allografts related to possible graft rejection, virus transmission and other ethical concerns [15, 16]. Similarly, xenografts may produce zoonotic disease in some cases [16]. These data may influence the opinions of clinicians and patients, and these opinions should determine the final treatment.

In general, the sociodemographic variables did not influence the refusal/acceptance rates of the various bone grafts options, and these results are consistent with those of a study by Hof et al. [17]. Similarly, no significant differences were observed in the acceptance/refusal rates for the different bone grafts according to religious affiliation. These findings may be attributable to the fact that only two religious branches were included in the sample, i.e., Catholic and Evangelical. These are the predominant religions of the Chilean population, and both allow the use of grafts derived from humans and animals [14]. Moreover, these findings demonstrate that the opinions about specific therapies are unique to each patient and not dependent on other factors.

Allografts were the bone grafts that elicited the highest refusal rate among respondents. 41 % declared that they would never accept this type of bone graft or would do so only as a last resort. A study involving 219 patients [6] who had received or were about to receive liver allotransplants reported on these patients' opinions about possible donor-related risks. Most patients wanted to be informed about the risk of infectious disease transmission (74.8 %). In the present study, 15 % of patients reported that they would refuse to accept a bone allograft due to fear of disease transmission from the donor.

Importantly, allografts are not available worldwide due to religious and ethical concerns [18]. However, in this study no significant differences were observed in the refusal rates for allographs due to specific religious affiliation. Moreover, despite the finding of a significant relation between women and allograft refusal, we cannot dismiss the possibility that this finding was influenced by the predominance of females in the sample. Despite the percentage of women not being representative of the total population at regional level, it is close to the percentage of females that receive attention in our clinics (68 % of patients). In relation to the percentage of religious/non-religious, data from the last Chilean census^1^ showed percentages of 67 % Catholic, 16 % Evangelical and 11 % non-religious. Therefore, this study exhibited percentages closer to the national population census with 55.5 % of individuals who declared themselves Catholic, 20.8 % Evangelical and 23.5 % non-religious.

Bone allografts from living donors are primarily obtained from the femoral head of patients undergoing hip-replacement surgery [19]. The donation criteria are quite strict, and over 50 % of willing donors are excluded. Accepted donors are tested for contagious diseases. Allograft bone from cadavers is also used, but in these cases information about the donor's lifestyle must be obtained from relatives, and this information may not always be reliable [19]. Nonetheless, the risks and benefits of allografts should be discussed with the patient prior to the consent process [15]. Such discussions are a part of the process of patient choice and essential to the principle of autonomy. Similarly, the clinician should be aware of the moral and ethical issues related to the use of allografts, the origins of the allografts, and the possible risks of disease transmission so as to respect the principles of beneficence and non-maleficence.

Regarding xenografts, 15 % of the patients reported that they would not accept this type of graft under any circumstance, and 18 % reported that they would approve of a xenograft only as a last resort. The main reasons for xenograft rejection were the fear of possible disease transmission and the belief that it is wrong to use animals for human benefit. Both of these opinions are valuable and must be considered by the clinician prior to the surgical procedure.

The ethical aspects of xenografts have also been a topic of discussion in the scientific community. Nelson [20] asked for the careful consideration of the ethical issues involved in the use of animal organs and the sacrifice of animal life for human benefit. McCarthy [21] reported that opponents of the use of animals may base their arguments on theological, philosophical and/or economic reasons. The rules and customs related to the use of products derived from animals may differ between different religions and individuals (Eriksson et al., 2013). For example, Hinduism does not allow the use of implants or products derived from cows or pigs, and Islam conflicts with the use of pig-derived products [14]. This study did not detect significant relationships between religion and xenograft primarily because Christian religions (i.e., Catholic and Evangelical) accept the use of animal-derived products [14]. In summary, some animal-derived products may cause conflicts with personal or religious beliefs that need to be considered prior to surgery.

In the case of autologous bone grafts, the majority of the patients who rejected their use provided reasons related to potential discomfort or postoperative pain at the donor site, whereas only 2 % mentioned religion. A study conducted by Nkenke and Neukam [22] reported that mandibular bone grafts are generally well-accepted procedures that also involve low objective and subjective morbidity rates. Banwart et al. [23] studied morbidity in 261 patients who had bone harvested from the iliac crest and concluded that serious complications can be avoided and those complications affecting the functions of the donor site were rare. In a study by Hof et al. [17] in which 150 patients were interviewed in relation to dental implants and bone grafts, 43 % chose a synthetic bone substitute material (alloplastic) to avoid donor site morbidity, and 23 % were able to undergo surgery to obtain an autograft from the hip. In the present study, only 8 % expressed that they would never accept the harvesting of an autologous bone graft from an extraoral donor site, for example, the tibia or hip.

Clinicians occasionally make decisions about the type of surgery and products used without concern for patient discussions, which violates the ethical principle of autonomy. Op den Dries et al. [6] found that 79.8 % of patients expressed the desire to be involved in decision-making regarding the advisability of accepting a liver transplant, 10.6 % wanted to make the final decision themselves, and only 9.6 % did not wish to be involved in the decision-making process. Therefore, the clinician must properly inform the patient without influencing his decision to obtain the patient’s opinion and informed consent for each product and procedure used in the treatment plan [14]. The final decision may depend on several factors, but it is necessary for the patient to be well informed [17]. In this sense, a professional must carefully consider the risks, costs and benefits of the type of bone graft to be used to achieve the final treatment outcome. Thus, the clinician must be sufficiently ethical and conscious of his duties to individuals and humanity.

One point that we wish to highlight is that despite the fact that all of the surveyed individuals were Christians, religion was one of the reasons for graft refusal. This finding was confusing for the authors but could be attributable to the differences that exist in individual religious interpretations [14]. Unfortunately, this question was answered without explanation. Both individuals that answered wrote: *“because the word of God says so”* and *“religious reasons”,* but without further explanation.

Little is known about patients’ opinions regarding bone grafts [17]; however, in this study we observed that each patient had a different opinion and strong arguments for choosing to accept or reject each type of bone graft, and this needs to be understood by the clinician prior to treatment planning. Unfortunately, it was not possible to directly compare previous studies with the present study because four types of bone grafts were examined here that have only recently become conventional. Similarly, this study employed a quantitative approach to the patients’ opinions about bone grafts. However, further studies employing qualitative analyses are needed to understand the different opinions in greater detail.

## Conclusions

Allografts and xenografts elicited the highest refusal rates among patients, and autologous bone and alloplastics were the most accepted types. Moreover, no differences in the acceptance/refusal rates for the different bone grafts due to the sociodemographic variables or religious affiliation were observed. Each patient expressed unique opinions and arguments for accepting or rejecting each type of bone graft. Therefore, although a clinician might have experience with a specific type of bone graft, he must ascertain the patient’s opinions before a treatment is planned.

## Footnotes

1 Available at http://www.ine.cl/canales/chile_estadistico/familias/censos.php.
